# (TCRαβ^+^) Double-Negative T Cells in Type 1 Diabetes Mellitus

**DOI:** 10.3390/cells15010058

**Published:** 2025-12-29

**Authors:** Dimitri Poddighe, Assel Mussayeva, Kuanysh Dossybayeva, Gulsamal Zhubanova, Dinara Galiyeva, Khac Linh Le, Matthew Naanlep Tanko

**Affiliations:** 1 College of Health Sciences, VinUniversity, Vinhomes Ocean Park, Gia Lam, Hanoi 100000, Vietnam; 2 School of Medicine, Nazarbayev University, Astana 010000, Kazakhstan; a.mussayeva@nu.edu.kz (A.M.); kuanysh.dossybayeva@nu.edu.kz (K.D.); matthew.tanko@nu.edu.kz (M.N.T.)

**Keywords:** double negative T cells, DNT cells, DN T cells, CD4^−^CD8^−^ T cells, NKT cells, autoimmune diabetes, diabetes mellitus type 1, NOD mice

## Abstract

**Highlights:**

**What are the main findings?**

- In murine autoimmune diabetes, TCRαβ^+^DNT cells appear to exert a predominantly protective role against immune-mediated β-cell injury.
- Very few studies have examined TCRαβ^+^DNT cells in patients with Type 1 Diabetes Mellitus (T1DM).

**What are the implication** **s of the main finding** **s?**

- TCRαβ^+^DNT cells might represent an additional therapeutic target in T1DM and other autoimmune conditions.
- Specific clinical and translational research is needed to better elucidate the role of TCRαβ^+^DNT cells in T1DM.

**Abstract:**

Type 1 Diabetes Mellitus (T1DM) is an autoimmune disease characterized by the destruction of pancreatic β-cells. Both lymphocytes and various innate immune cells contribute to its immunopathogenesis. Among lymphocytes, in addition to CD8^+^ T cells, CD4^+^ T cells, and B cells, growing attention has been directed toward some unconventional T-cell subsets, such as TCRαβ^+^ double-negative T (DNT) cells, based on findings in several autoimmune/rheumatic diseases. This narrative review aims to summarize and analyze the available data on the potential role of DNT cells (and, in detail, the TCRαβ^+^ subset) in the immunopathogenesis of autoimmune diabetes/T1DM. Most of the current knowledge regarding DNT cell homeostasis in this pathological setting derives from experimental models, especially Non-Obese Diabetic (NOD) mice. In murine autoimmune diabetes, TCRαβ^+^DNT cells appear to exert a predominantly protective role against immune-mediated β-cell injury. These cells can be observed in multiple anatomical sites, including the thymus, peripheral blood, secondary lymphoid organs (spleen and lymph nodes) and, under pathological conditions, in non-lymphoid organs, like within the pancreas and, in detail, pancreatic islets, in the setting of autoimmune diabetes. Experimental evidence suggests that TCRαβ^+^DNT cells may attenuate the CD8^+^ T cell-mediated destruction of pancreatic β-cells, both directly and indirectly, through the inhibition of CD4^+^ T cells and B cells implicated in this immunopathological process. Unfortunately, very few studies have examined TCRαβ^+^DNT cells in patients with T1DM. This important knowledge gap highlights the need for dedicated clinical and translational research to better elucidate the role of TCRαβ^+^DNT cells in T1DM, especially given the preliminary findings pointing toward their potential immunoregulatory relevance.

## 1. Introduction

Type 1 Diabetes Mellitus (T1DM) is an autoimmune disease characterized by the destruction of β-cells within the pancreatic islets. This immune-mediated process progressively leads to the loss of insulin production, ultimately resulting in complete dependence on insulin replacement therapy [1,2].

Globally, Diabetes Mellitus (DM) represents one of the major non-communicable diseases with a profound medical burden and substantial economic impact. Although T1DM (now accounting for <5% of all DM cases) is far less prevalent than Type 2 Diabetes Mellitus (T2DM), it poses considerable challenges, particularly in children and young adults. Affected individuals face lifelong reliance on exogenous insulin and, due to a longer life span, an extended period at risk for DM-related chronic complications, such as retinopathy, nephropathy, neuropathy, and cardiovascular disease [3,4,5].

Green et al. estimated the global prevalence and incidence of T1DM across all age groups. According to their model and calculations, the global numbers of incident and prevalent cases of T1DM in 2017 were 234,710 and 9,004,610, respectively. Although T1DM incidence peaks during the pediatric age, the global prevalence was 6%, 35%, 43%, and 16% in the age groups 0–14, 15–39, 40–64, and >65 years, respectively, which further underscores the lifelong and general impact of T1DM [6].

The diagnosis of T1DM generally relies on the detection of “islet autoantibodies”, in addition to the evidence of altered glycemic homeostasis. According to the American Diabetes Association (ADA) Standards of Care in Diabetes, “glutamic acid decarboxylase (GAD) autoantibody should be measured and, if negative, should be followed by islet tyrosine phosphatase 2 (IA-2) and/or zinc transporter 8 (ZnT8)”, when available [7].

At the time of the diagnosis, most T1DM patients have at least one (or more) positive antibody [8]. This positivity to “islet autoantibodies” highlights the main immunopathogenic aspects of T1DM. Although the initial triggers of β-cell-directed autoimmunity in (genetically) susceptible individuals has yet to be fully elucidated, and both humoral and cell-mediated immune mechanisms are involved, the production of specific autoantibodies (e.g., GAD, IA-2, ZnT8, and others) serves as a biomarker for this autoimmune process (and, thus, for supporting the final diagnosis of T1DM, as mentioned), but these are considered as “non-pathogenic” autoantibodies [9,10].

Extensive studies have advanced our understanding of T-cell-mediated destruction of β-cells in T1DM. The hallmark pathological finding observed in pancreatic tissue from individuals with recent-onset T1DM is a patchy and variable degree “insulitis”, defined as infiltration of pancreatic islets by inflammatory/immune cells. Notably, this infiltrate is predominantly composed of T cells but also includes B lymphocytes, neutrophils, macrophages, and other immune cells. Both CD4^+^ and CD8^+^ T cells simultaneously infiltrate the pancreatic islets, with the former ones typically predominating [9,11,12]. These T cells can recognize a multitude of islet autoantigens and, within a single inflammatory infiltrate, T cells with different autoantigen specificity can coexist, especially in patients with longer disease duration [13,14]. Whereas CD8^+^ T cells (directly) attack the β-cells and, thus, determine their destruction, CD4^+^ T cells are supposed to orchestrate this immunopathological process [15].

However, many other (adaptive and innate) immune cells also contribute to this complex autoimmunity process [16]. Although the “anti-islets autoantibodies” themselves are non-pathogenic, B cells are supposed to promote the β-cell destruction by serving as antigen-presenting cells for autoreactive T cells. Indeed, B cells producing these autoantibodies can present peptides derived from their target antigens to T cells, and antigen–antibody complexes can be internalized by dendritic cells, enhancing the cytotoxic activity of CD8^+^ cells. Notably, the B-cell depletion with rituximab at T1DM onset has been shown to delay the β-cell (and insulin) loss [17,18,19].

Among non-lymphoid cells, neutrophils infiltrating the pancreatic islets have been suggested to contribute to tissue injury by secreting proinflammatory cytokines and chemotactic factors, in general. Evidence also suggests that neutrophils may even be involved in the early stages of the autoimmune response, influencing both innate and adaptive immunity and modulating the inflammatory micro-environment of the pancreatic islets. Their interactions with other immune cells involve multiple mechanisms, including the secretion of mediators, phagocytosis, production of reactive oxygen species, and release of Neutrophil Extracellular Traps (NETs), which may increase the exposure of self-antigens to the adaptive immune system [20,21,22]. Additionally, Natural Killer (NK) cells could participate in several stages of T1DM immunopathogenesis. Evidence showed that NK cells can also infiltrate the pancreatic islets and that, through the high expression of some activating receptors, they may exert their cytotoxicity against the pancreatic β-cells [23]. Indeed, the imbalance among distinct subpopulations of tolerogenic and cytotoxic NK cells may be implicated in the immune dysregulation and also play a role in the potential and proposed involvement of viral infections as triggers of T1DM autoimmunity [24].

Recently, growing attention has focused on minor or “unconventional” T-cell subsets, such as Double-Negative (CD4^−^ and CD8^−^) T cells (DNT cells), due to their emerging regulatory role and their implication in several autoimmune diseases [25,26]. This review aims to summarize and analyze the current evidence and perspectives related to a potential role of DNT cells (with a specific focus on TCRαβ^+^DNT cells) in the immunopathogenesis of T1DM. To frame the scope of this narrative review, we conducted a non-systematic literature search across PubMed, using combinations of terms related to (TCRαβ^+^) DNT cells and autoimmune diabetes/T1DM. Additional relevant publications were identified through reference screening and expert knowledge of the field. The selection of articles was guided by conceptual relevance and the contribution of each work to current understanding, rather than by predefined inclusion or exclusion criteria. This approach ensured a comprehensive yet context-driven synthesis of available evidence, consistent with the narrative nature of this review.

## 2. (TCRαβ+) Double-Negative T Cells

DNT cells are a small subset of T lymphocytes (<2% of circulating lymphocytes) characterized by the absent expression of both CD4 and CD8 markers, while expressing the T-cell receptor (TCR), which can be composed of either αβ or γδ chains [27,28].

Broadly speaking, based on the expression analysis of these three aforementioned T-cell markers, NKT cells can appear immuno-phenotypically similar to DNT cells. However, NKT cells are distinguished by their concomitant expression of NK-associated markers (such as CD16, CD56, and CD161), which are not present on αβTCR^+^ or γδTCR^+^ DNT cells. NKT cells also express a TCR, but this is characterized by a restricted specificity [29]. NKT cells can be further subdivided into type I and II. Type I NKT cells are also known as “invariant” NKT (iNKT) cells, since they express a specific CD1d-restricted semi-invariant TCR that specifically recognizes the lipid antigen galactosyl-ceramide (GalCer). Type II NKT cells express a different but still limited TCR repertoire, allowing recognition of a broader set of CD1d-presented glycolipids and phospholipids; however, their antigen specificities remain incompletely defined yet [30,31]. NKT cells also play a role in the immunopathogenesis of T1DM. These cells may exert an immunoregulatory action and, in the context of T1DM, they are thought to have a protective role: impaired NKT cell function may predispose to pancreatic β-cell autoimmunity by reducing peripheral control of autoreactive cytotoxic CD8^+^ T cells [32,33,34].

Returning to the discussion on TCRαβ^+^DNT cells (which represent the core topic of the present review), recent evidence suggests that these cells can be generated through both thymus-dependent (by escaping from the negative selection process) and thymus-independent pathways. In the latter case, under specific immunological situations, activated peripheral lymphocytes could lose the expression of their CD4 or CD8 markers, but the exact ontogeny of these cells remains incompletely defined [26,35]. This possibility is supported by several murine and human studies, particularly in autoimmunity settings [25]. Through a series of experiments in mice, Rodriguez-Rodriguez et al. concluded that a substantial portion of peripheral TCRαβ^+^CD4^−^CD8^−^ DNT cells derive from CD8^+^ T cells rather than from CD4^+^ T cells; under certain conditions (e.g., lymphocyte chronic activation, lymphopenia, autoimmunity), some CD8^+^ T cells may downregulate CD8 and convert their phenotype into DNT cells, which may persist in secondary lymphoid organs and expand upon specific stimulations [35]. Consistent with this, Crispin et al. previously showed that the gene expression pattern analysis of TCRαβ^+^DNT cells displays more similarities with CD8^+^ lymphocytes than CD4^+^ T cells [36]. In humans, Bristeau-Leprince et al. observed similar TCR Vα and Vβ usage between CD8^+^ T cells and DNT cells in patients with Autoimmune LymphoProliferative Syndrome (ALPS) [37].

Notably, TCRαβ^+^DNT lymphocytes are markedly expanded in the context of ALPS, where a TCRαβ^+^DNT cell count >1.5% of total lymphocytes and/or >2.5% of CD3^+^ lymphocytes represents one of the diagnostic criteria [38]. Overall, ALPS is caused by genetic alterations affecting FAS signaling. In detail, germline and/or somatic pathogenic mutations in genes, such as FAS, FAS ligand, or FADD, can be causative for ALPS [39]. Several and different types of autoimmune manifestations can occur in ALPS patients [38], and this aspect has prompted broader investigations on (TCRαβ^+^) DNT cells as a potential contributor to autoimmunity in general. The expansion of the (TCRαβ^+^) DNT cell pool was reported and/or suggested in several immune-mediated diseases, particularly in Systemic Lupus Erythematosus [25,27,29].

This particular interest in investigating TCRαβ^+^DNT cells in SLE is partly explained by the fact that one of the main murine experimental models for lupus is represented by the MRL/lpr mouse, which is characterized by the mutation in the lpr gene encoding the FAS receptor. Indeed, in addition to developing a SLE-like phenotype (including the occurrence of nephritis and high titers of autoantibodies such as ANA and anti-dsDNA), this mouse also shows a strong lymphoproliferation sustained by the accumulation of DNT cells [40,41]. However, studies examining DNT cell frequency and function in SLE and other rheumatic diseases have yielded conflicting results regarding their expansion and potential contribution to disease pathogenesis [25,28,41,42,43].

## 3. (TCRαβ^+^) DNT Cells in Autoimmune Diabetes (Insights from Experimental Models)

To our knowledge, the first study suggesting the potential implication of DNT cells in autoimmune diabetes (as an experimental surrogate of T1DM in humans) dates back to 1990, when Chandy et al. published a study describing an increased expression of an unusual form of K^+^ channel, namely type I, in DNT cells (CD4^−^CD8^−^Thy1.2^+^) from four different murine experimental models of specific autoimmune diseases, namely NZBxNZWF1 and MRL^+/+^ (for lupus), NOD (for autoimmune diabetes), and SJL/PLJ (for experimental allergic encephalomyelitis) mice. Specifically in NOD mice, splenic DNT cells from overtly diabetic mice displayed an increased number of type I K^+^ channels (around 200/cell), which was comparable to the findings in the other two diseases and significantly higher than in control mice [44].

Although more recent studies have explored the possibility that these K^+^ channels may play a role in the function of T cells [45,46], subsequent research has not specifically addressed this question in DNT cells and provided no additional evidence supporting their involvement in autoimmunity or their utility as disease markers in autoimmune diabetes. Nonetheless, this and other studies by Chandy’s group drew attention to DNT cells in NOD mice and, more generally, in autoimmune diabetes [44,46,47,48].

Formby et al. later investigated the characteristics of pancreatic islet inflammatory infiltrate in this mouse model and showed that DNT cells constituted a substantial proportion of the infiltrating T-cell population (approximately 20%). Interestingly, DNT cells were proportionally increased in the spleen to a similar degree [49].

Comparable findings were obtained in rat models: diabetes-prone and acutely diabetic BB rats exhibited increased splenic DNT cell percentages compared with diabetes-resistant BB rats and normal strains (Wistar-Furth). Notably, in this study, such an increase in DNT cells was specifically attributed to the TCRαβ^+^DNT cell subset [50].

Beyond the spleen and pancreatic islets, increased numbers of DNT cells in NOD mice were also reported in the thymus, especially in long-term diabetic mice, which was accompanied by a reciprocal depletion of the “double positive” T cells [51,52]. However, other authors reported conflicting findings regarding the dynamics of thymic DNT cells [53]. However, the analysis of DNT lymphocyte precursors in this primary lymphoid organ (and, thus, during lymphopoiesis and T-cell precursor maturation) is beyond the specific aims of the present analysis, which is focused on the homeostasis and features of the peripheral pool of DNT cells.

Goldrath et al. conducted a comprehensive analysis of lymphocytes in islet infiltrates, pancreatic lymph nodes, and peripheral blood of NOD mice, demonstrating that the proportion of infiltrating DNT cells was significantly higher than that observed in peripheral blood or lymph nodes [54].

Up to this point, all the available studies have focused mainly on phenotypic and/or quantitative analyses of DNT cells in different tissues from NOD mice. In 2007, Ford et al. tried to investigate whether DNT lymphocytes (precisely identified as CD3^+^CD4^–^CD8^–^NK1.1^–^TCRαβ^+^ cells) could be “functionally” involved in the onset of diabetes. They used a specific experimental model wherein autoimmune diabetes was induced by the gp33–41 peptide in P14/RIP-gp mice (P14 mice express a transgenic TCR specific for the gp33–41 peptide presented in the context of MHC class I; RIP-gp mice express gp33–41 on the pancreatic β-cells under the control of the rat insulin promoter). Briefly, their experiments suggested that TCRαβ^+^DNT cells can be activated by peptides presented in the context of self MHC in an antigen-specific fashion, and that they can suppress and kill peptide-activated syngeneic CD8^+^ T cells. Thus, TCRαβ^+^DNT cells were considered to be able to potentially inhibit the development of autoimmune diabetes, as further supported by the fact that the infusion of peptide-activated DNT cells was able to reduce its occurrence. Therefore, in this experimental model, DNT cells clearly appeared to act as regulatory and/or suppressor cells of (autoreactive) CD8^+^ T cells [55].

Mohamood et al. investigated the onset of diabetes and its relationship with FasL mutations by using bred NOD-gld/gld mice. In addition to showing that FasL expression plays a direct role in the pathogenesis of autoimmune diabetes in this experimental system, they concluded that the protection from autoimmune diabetes induced by gld mutation (of the FasL gene) is genetically dissociable from gld-induced DN T-cell lymphoproliferation [56]. As relevant background for this study, it is important to mention earlier studies showing that high levels of Fas and FasL are expressed by β-cells in the pancreatic islets from T1DM patients, and that Fas defects could delay the disease onset in the NOD experimental models, even despite the occurrence of autoimmunity/lymphoproliferative phenomena, as typically described in MLR lpr/lpr and gld/gld mice and patients with the ALPS (where the inherited loss-of-function mutations of the Fas/FasL genes is present and the expansion of TCRαβ^+^DNT cells is an important marker, as explained in the previous section) [57,58,59].

Collectively, studies up to the early 2000s revealed the complexity and heterogeneity of the potential implications of TCRαβ^+^DNT cells in autoimmune diabetes, possibly reflecting the existence of distinct DNT cell subsets. Nonetheless, the aforementioned protective role of these DNT cells against autoimmune diabetes has also been suggested by other experimental findings. For instance, the results in the study by Duncan et al. showed that splenic TCRαβ^+^DNT cells from young NOD mice conferred long-lasting protection against autoimmune diabetes. In detail, this protective “antidiabetogenic” population was precisely described as CD3^+^CD4^−^CD8^−^CD28^+^CD69^+^CD25^low^Foxp3^−^iCTLA-4^−^TCRαβ^+^ cells [60].

A notable study by Dugas et al. included CD47-deficient mice (CD47^−/−^), which were also prone to autoimmune diabetes. CD47 is also known as the “integrin-associated protein”, and it is a transmembrane protein implicated in immune regulation through its interactions with members of the Signal Regulatory Protein (SIRP) family (especially SIRPα). CD47 deficiency resulted in influencing the homeostasis of DNT cells in the peripheral lymphoid organs and, in detail, the reduction in DNT cells (in the spleen and lymph nodes) correlated with the occurrence of diabetes. Notably, the passive transfer of these DNT cells could inhibit the onset of the disease by restoring the immune tolerance [61]. In a subsequent study, the same research group confirmed the importance of DNT cells in terms of immunoregulatory activity in the NOD mice. In detail, they confirmed the immunoregulatory properties of DNT cells in NOD mice, showing that they possess cytotoxic activity toward activated B cells and that IL-10 can limit their expansion and function by inducing apoptosis [62].

The inhibitory effect of DNT cells in NOD mice is also directed to conventional T cells, especially CD8^+^ T cells (but CD4^+^ T cells as well), both in vivo and in vitro. Liu et al. described the reversion of autoimmune diabetes in NOD mice treated with DNT cells along with anti-thymocyte serum; the isolated use of DNT cell transfer could only postpone its occurrence. Zhang et al. further demonstrated that the adoptive cell therapy with β-cell antigen-specific DNT cells (induced from prediabetic NOD CD4^+^ T cells in vitro) could protect against the development of autoimmune diabetes [63,64]. 

The association between an increased proportion of DNT cells in lymphoid organs and a decrease in diabetes incidence and autoantibody serum levels was also confirmed by Collin et al. In this study, an additional finding was that the *Idd2* locus (which is implicated in the genetic susceptibility to autoimmune diabetes) was linked to DNT cell proportion [65]. However, other genetic regions (e.g., *Idd9* and *Idd13*) in NOD mice can be implicated in the DNT cell regulation, also starting from the thymic development, where the maturation through the “double negative” stage of the thymocyte can be impaired [66,67,68].

More recently, Islam et al. performed a longitudinal single-cell RNA-sequencing analysis of peripheral blood T cells in NOD mice, demonstrating progressively elevated levels of circulating and islet-infiltrating DNT cells, accompanied by exhaustion of their “immunosuppressive” subsets. This shift apparently contributed to clonal expansion of CD8^+^ T cells infiltrating the pancreas [69].

In general, despite certain limitations (including an incomplete immunophenotype analysis in several studies), all these research efforts (summarized in Table 1, which provides a schematic summary of the main methodological aspects and main findings from each individual study) overall suggest and/or support a role for TCRαβ^+^DNT cells in the immunopathogenesis of autoimmune diabetes.

## 4. (TCRαβ^+^) DNT Cells in Diabetes Mellitus Type 1: Knowledge Gaps and Perspectives

Unfortunately, very few studies have investigated (TCRαβ^+^)DNT cells in patients affected with T1DM.

One of the most relevant and recent contributions is the study by Barcenilla et al. [70]. This study should be considered in the landscape of a series of research projects aiming to explore the antigen-specific immunotherapy (based on the subcutaneous administration of glutamic acid decarboxylase 65, GAD65) as a strategy to preserve the β-cell function in patients with T1DM [71,72,73,74]. In the aforementioned study, Barcenilla et al. further investigated several immunological aspects and responses following the intra-lymphatic administration of GAD65 along with aluminum hydroxide (GAD-alum), in order to try improving the efficacy of this therapeutic approach (compared to the subcutaneous route) [70], which resulted in being safe and tolerable in a previous open-label clinical trial [75,76]. In detail, the authors observed an expansion of a CD69^+^PD-1^+^DNT cell subset after GAD-alum treatment. These DNT cells may derive from CD8^+^ T cells upon antigen encounter, may be self-reactive, and may upregulate the expression of PD-1. Thus, this DNT cell expansion could reflect reduced activation of CD8^+^ T cells and a diminished proliferative response to GAD65. However, this study does not provide any specific and/or direct evidence supporting an immunopathogenic role of DNT cells. Moreover, the definition of DNT cells used in the methods of this article (“CD3+CD4−CD8−”) did not specify TCRαβ expression, leaving open the possibility that NKT cells could have been included in this population, too [70].

A more recent study by Fajardo-Despaigne et al. aimed to characterize the TCRαβ^+^DNT cell subpopulation in peripheral mononuclear blood cells from T1DM patients. They enrolled 16 adults (age range: 18–37 years) with T1DM and 11 controls. In quantitative terms, the DNT cells (which were precisely defined as TCRαβ^+^CD8^−^CD4^−^CD56^−^) were <2% in both T1DM patients and healthy controls. No difference in TCRαβ^+^DNT cells was observed between these groups of patients in terms of percentage (of total T cells) or absolute cell count. However, the additional value of this study is that TCRαβ^+^DNT cells were characterized in terms of both transcriptomic and protein analysis. Although no major phenotypical and/or functional differences between TCRαβ^+^DNT cells (and conventional T cells, too) derived from T1DM patients and controls were observed, two broader observations (that could even be applied to other autoimmunity settings) emerged from this study [77]. First, TCRαβ^+^DNT cells shared many similarities with conventional CD8^+^ T cells, which would be consistent with the main hypothesis that TCRαβ^+^DNT cells (or at least a portion of them) mainly originate from CD8^+^ T cells after downregulation or loss of the CD8 molecule in response to chronic immune activation or inflammatory conditions, as supported by a number of previous studies [78,79,80]. Second, blood-derived TCRαβ^+^DNT cells can be efficiently expanded in vitro by using different cytokine combinations, generating a large and pure pool of DNT cells for potential therapeutic applications. Indeed, these DNT cells seem to keep most of their original functional properties, and therefore, this study could provide an important rationale and background to consider the development of DNT cell-based therapy for T1DM and perhaps other autoimmune diseases [77].

Very recently, DNT cells and, more precisely, one subset (“EM”, effector memory) with specific immunophenotypic characteristics, were mentioned in a study exploring the immunopathogenesis of T1DM by Mendelian randomization. Through this approach, the main conclusion of this study was that CD28^+^CD45RA^+^CD8^bright^ cells (and, in detail, CD28 expression on this subpopulation) were “significantly” associated with genetic susceptibility to T1DM, while “EM” DNT cell percentage (like other subsets) could be “potentially” associated with genetic susceptibility to T1DM [81]. Unfortunately, the complete immunophenotypic definition of these EM DNT cells was not provided in the Methods Section of this article.

Therefore, human studies examining the homeostasis and functional roles of TCRαβ^+^DNT cells in T1DM are still in their infancy. This knowledge gap warrants further investigation, especially given that this cellular population may participate in key immunoregulatory pathways and could represent an additional therapeutic target in T1DM and other autoimmune conditions.

Indeed, more broadly (beyond T1DM), accumulating evidence from both murine and human studies indicates that TCRαβ^+^DNT cells can exert immunoregulatory functions with a therapeutic potential across different and multiple pathological settings [82,83,84]. For instance, in graft-versus-host disease (GvHD), DNT cells were reduced in patients at the initial stage of the chronic forms, and human DNT cells were able to delay the onset of xenogeneic GvHD in humanized mouse models [85,86]. As discussed earlier in the context of some of the aforementioned studies [26,56,57], DNT cells have been reported to mediate immune suppression via Fas-FasL interactions, as well as through perforin/granzyme secretion or modulation of dendritic cells and other antigen-presenting cells [87,88]. Interestingly, Haug et al. found that human “regulatory” DNT cells could not only suppress the proliferation of effector T cells but also alter some metabolic and functional characteristics, suggesting a role of DNT cells in promoting the peripheral tolerance after allo-HSCT and, thus, supporting their potential use as cellular “immunoregulatory” therapy, in this case to prevent or diminish GvHD [89]. More recent work even suggested that the additional value in this context could also be a graft-versus-leukemia (GVL) activity, in addition to reducing the GvHD [90,91,92]. Notably, several studies also noted that persistent CAR-T cells in patients achieving long-term remission predominantly display a DNT phenotype [91,93,94,95]. Recently, CAR-T cell therapy has also been considered in the setting of different autoimmune diseases with a severe clinical course, particularly in SLE and also in pediatric patients [96,97,98,99,100]. T1DM could also be considered in perspective [101,102]. However, in the setting of CAR-T cell therapy for autoimmune diseases (and specifically for T1DM), the specific “immunoregulatory” role and value of TCRαβ^+^DNT cells remains completely unexplored so far.

In summary, current human studies provide only a fragmented understanding of how TCRαβ^+^DNT cells behave in the context of T1DM. Most available analyses rely heavily on peripheral blood sampling, which captures only a small fraction of the relevant immunobiology and overlooks tissue-resident or pancreas-draining lymph node populations that are likely central to T1DM pathogenesis. Furthermore, phenotypic characterization often lacks functional correlates, such as suppressive capacity, metabolic status, or antigen specificity, which should also be explored in the specific context of T1DM. Several limitations in the existing datasets (e.g., small cohort sizes, variable therapeutic history, limited or no longitudinal follow-up), that collectively hinder the ability to draw causal relationships or define reproducible immunological signatures, should also be addressed in future and focused studies. Finally, the lack of standardized methodologies for identifying and/or quantifying specific subsets of DNT cells added yet another layer of variability across the existing studies. Together, all these limitations should be considered in the future and prospective research efforts to clarify the role of TCRαβ^+^DNT cells in T1DM and, in general, different autoimmune diseases.

## 5. Conclusions

Although the TCRαβ^+^DNT cell pool represents a very small and unconventional subset of T cells, it likely comprises several and different cellular subpopulations with specific and distinct immunophenotypic and functional (both inflammatory and regulatory) properties, which may be variably represented and implicated across different pathological settings.

**Figure 1:**
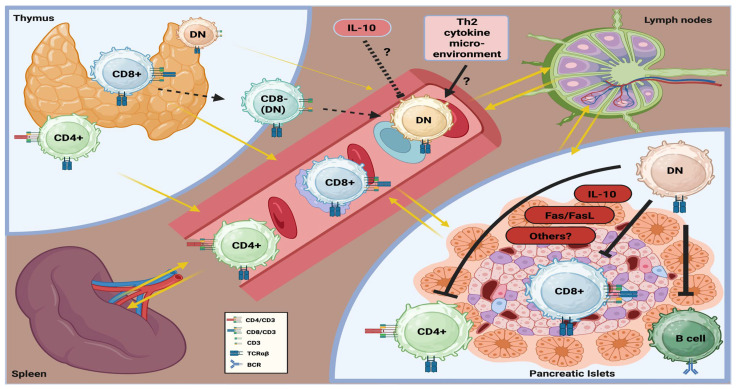
Graphical summary of the homeostasis and potential role of TCRαβ^+^DNT cells in the immunopathogenesis of autoimmune diabetes.

As graphically summarized in Figure 1, evidence from murine autoimmune diabetes (and, perhaps, in human T1DM) suggests that TCRαβ^+^DNT cells may exert immunoregulatory functions and protect β-cells from the immune-mediated injury. These cells have been detected in several anatomical sites, including the thymus, peripheral circulation, secondary lymphoid organs (spleen and lymph nodes), and non-lymphoid organs, such as the pancreas (and specifically within the pancreatic islets), in the setting of autoimmune diabetes. Indeed, experimental findings suggested that TCRαβ^+^DNT cells could reduce the CD8^+^ T cell-mediated destruction of pancreatic β-cells directly and also indirectly, by inhibiting both CD4^+^ T cells and B cells implicated in this immunopathological process. Their immunoregulatory activity appears to rely on multiple molecular mechanisms (which are still not fully defined), including the release of cytokines (such as IL-10), Fas/FasL signaling pathway, and other (contact) cellular interactions. Similarly to other autoimmune diseases, TCRαβ^+^DNT cells are likely to mainly derive from CD8^+^ cells after peripheral loss of this molecule, rather than thymic maturation directly. However, factors driving their expansion and function remain poorly understood. Some experimental models suggest that a Th2-skewed cytokine environment may promote their development, whereas cytokines such as IL-10 may inhibit it. Most (almost all) of these initial observations originate from murine models of autoimmune diabetes (primarily NOD mice). Therefore, additional experimental studies specifically addressing TCRαβ^+^DNT cells, as well as clinical studies exploring their homeostasis and response in humans, are definitely needed to elucidate their role in the immunopathogenesis of T1DM and their potential therapeutic relevance, if any.

## Figures and Tables

**Table 1:** Summary of the main studies providing information on DNT cells in experimental models of autoimmune diabetes.

First Author, Year, Country [Ref.]	Biological Samples	Main Autoimmune Diabetes Model	DNT Cell Markers	Study Aim	Main Analytical Methods and Equipment	Additional Findings on DNT Cells	Main Conclusion
Chandy et al., 1990, USA [44]	Spleen	NOD	CD4^−^ CD8^−^ Thy1.2^+^	To assess the voltage-gated K^+^ channel expression in T cells and, in detail, DNT cells	FACS [n/a] Patch-clamp whole-cell recordings Fluorescence microscopy	--	*“…augmented type l K+ channel expression* *appears to be a valuable marker for DN T cells associated with murine lupus, autoimmune mellitus and experimental autoimmune encephalomyelitis”*
Zipris et al., 1991, Canada [51]	Lymph nodes Thymus	NOD	CD4^−^ CD8^−^	To assess the pattern of thymic T-lymphocyte development and the expression of the peripheral T-lymphocyte repertoire before and at the onset of diabetes	FACS [EPICS V flow cytometer (Coulter)]	*“Prediabetic NOD mice displayed an increase in CD4^−^CD8^−^ T thymocytes, and a reciprocal decrease inCD4+CD8+ T thymocytes. The CPM-accelerated diabetes was characterized by an increase of DN thymocytes in the days following the injection.PS. No information described about peripheral DNT cells.”*	*“… depletion of CD4+ regulatory T* *lymphocytes and/or the rerouting of CD4+V8.1+ effector T lymphocytes from the peripheral LN to the pancreas during progression to disease onset mediate the pathogenesis of diabetes.”*
Formby et al., 1992, USA [49]	Spleen Pancreas (islets)	NOD	CD4^−^ CD8^−^ Thy1.2^+^	To perform a quantitative analysis and functional assessment of the inflammatory cells	FACS [n/a (Becton Dickinson)]	--	*“In summary our results show that isolated prediabetic* in situ *islet immune cells are mostly CD4 positive and double negative T cells.”*
Hosszufalusi et al., 1992, USA [50]	Spleen	[rats] DP	CD4^−^ CD8^−^ αβTCR^+^	To perform a systematic FACS analysis of all major spleen cell populations	FACS [FACScan (Becton Dickinson)]	--	*“… autoimmune diabetes may reflect an immune balance abnormality with a relative shift of cell subset dominance toward double-negative or activated T cells, NK cells and macrophages.”*
Zhang et al., 1994, China [52]	Spleen Lymph nodes [Thymus]	NOD	CD4^−^ CD8^−^ Thy1.2^+^	To detect any immunological alterations that can precede the onset of autoimmune diabetes	FACS [FACScan (Becton Dickinson)]	*“The most striking observation was the increase in the proportion of CD4 and CD8 double-negative thymocytes in the long-term diabetics despite the fact that their absolute number was fairly.” PS. No information on DNT cells in peripheral tissues.”*	*“Our studies indicate a gradual absolute increase, or at least, a sustained percentage increase of CD8+ T cells in both the spleen* *and pancreatic LN of female NOD mice compared to CD4+T cells which gradually decrease.”*
Goldrath et al., 1995, USA [54]	Blood Lymph nodes Pancreas (islets)	NOD	CD4^−^ CD8^−^ [αβTCR] [γδTCR]	To characterize the immunophenotype of infiltrating lymphocytes isolated from the islets and compare these phenotypes to those of peripheral lymphocytes	FACS [FACScan (Becton Dickinson)]	*“In this study we also find a significant increase in the number of CD3+CD4^−^CD8^−^ double-negative cells in the infiltrating as compared with the peripheral T cell population (11–17%* vs. *less than 5% in the periphery).”*	*“Significantly increased levels of CD4+CD8+ double-positive and CD4^−^CD8^−^ double negative T cell populations were observed in the infiltrating lymphocytes as compared with peripheral lymphocytes. In addition, within both CD4 and CD8 subpopulations isolated from islet infiltrates, CD11b+ and CD49e+ (adhesion markers) cells were increased with respect to the same subset of cells isolated from the periphery. In contrast, the level of cells that expressed L-selectin was significantly higher in the periphery for both CD4+ and CD8+ cells than for infiltrating cells.”*
Ford et al., 2007, Canada [55]	Spleen Lymph nodes	P14 RIP-gp P14/RIP-gp	CD4^−^ CD8^−^ Thy1.2^+^ Vα2^+^ NK1.1^–^	To assess the capacity of DNT cells to recognize peptides expressed on self-MHC, to suppress autoreactive CD8+ T cells	MS magnetic column (Miltenyi Biotech)	--	*“In summary, the data presented here demonstrate that DN T cells are activated by peptides presented in the context of self MHC in an antigen-specific fashion and that they can suppress and kill peptide-activated syngeneic CD8+ T cells.”*
Mohamood et al., 2007, USA [56]	Bone marrow Gut epithelia “Immune-privileged sites”	NOD	CD4^−^ CD8^−^ αβTCR^+^	To investigate the role of FasL and DNT cells in autoimmune diabetes	FACS [n/a]	*“In addition, we demonstrate genetically, in bone marrow chimeras and haploinsufficient NOD-gld/mice, and pharmacologically, using FasL-neutralizing antibody, that the protective effect of FasL inactivation can be achieved without causing DN* *T-cell lymphoproliferation”*	*“…we show that FasL expressed on hematopoiet and nonhematopoietic compartments plays nonredundant roles in the pathogenesis of autoimmune diabetes. Mutation of FasL in either compartment interferes with the autoimmune process and prevents onset of diabetes. Moreover, FasL expressed in the hematopoietic compartment is the dominant regulator of T-cell homeostasis.*
Duncan et al., 2010, USA [60]	Spleen	NOD	CD4^−^ CD8^−^ αβTCR^+^	To analyze the function and phenotype of DNT splenic cells	FACS [FACSAria cell sorter (BD Biosciences)] RT-PCR (IL-10 analysis)	*“Their [DNT cells’] suppressive (antidiabetogenic) effect relied mainly on the ability to differentiate into IL-10-secreting TR-1 cells in a Th2-like extra-thymic environment.”*	*“…this study delineates a new cell population of regulatory cells (DNCD3 [Double negative CD3+4^−^8^−^ TCRαβ splenic cells]) in young NOD mice with potential anti-diabetogenic effect. The phenotype of DNCD3 splenic cells is CD3+ (CD4^−^CD8^−^)CD28+CD69+CD25lowFoxp3-iCTA-4^−^ TCRαβ+) (anti-diabetogenic phenotype) with a predominant Vβ13 gene usage.”*
Dugas et al., 2010 Canada [61]	Spleen Lymph nodes	NOD	CD4^−^ CD8^−^ B220^−^ CD5^low^ CD1d- tetramer^-^ βTCR^+^	To assess the contribution of the CD47 pathway in autoimmune diabetes.	FACS [FACSVantage (BD Biosciences)]	*“Decreased proportion of CD4^−^ CD8^−^ T cells in CD47-deficient mice”*	*“In summary, our observations have permitted the association of a defect in the CD47 pathway with autoimmune diabetes progression and identify at least part of the mechanism by which the disruption of the CD47 pathway accelerates disease onset; through the regulation of DN T cell number.”*
Hillhouse et al., 2010, Canada [62]	Spleen	NOD	CD4^−^ CD8^−^	To assess whether the activity of DNT cells is impaired in autoimmune diabetes	FACS [FACSCalibur, FACS LSR (BD Biosciences)] FlowJo	--	*“On the basis of these results, we conclude that, on a per cell basis, the DN T-cell cytotoxic function is not impeded in the autoimmune-prone NOD genetic background”*
Zhang et al., 2011, USA [64]	Spleen Lymph nodes	NOD	CD4^−^ CD8^−^	To study whether DNT cells generated from NOD mice retain the antigen-specific regulatory capacity and prevent autoimmune diabetes in vivo	FACS [FACSAria (BD Biosciences)]	--	*“In short, this study demonstrates that beta cell antigen-specific DNT cells can be induced from prediabetic NOD CD4+T cells* in vitro, *efficiently prevent the onset and progress of autoimmune diabetes* in vivo, *and also act in conjunction with rapamycin to promote islet allograft survival in NOD mouse models. “*
Dugas et al., 2014 Canada [66]	Spleen Lymph nodes	NOD	CD4^−^ CD8^−^	To assess the impact of major insulin-dependent diabetes (Idd) loci on the number of DNT cells	FACS [FACSCalibur (BD Biosciences)]	*“Together, our results show that the regulation of DN T-cell number in NOD mice is at least partially conferred by alleles at the Idd13 locus”*	*“In conclusion, at least five traits are linked to Idd13, namely, the degree of insulitis, thymic selection as well as the number of merocytic dendritic cells (mcDCs), NKT and DN T cells. […] it is likely that at least two genes within the Idd13 locus contribute to diabetes susceptibility.”*
Collin et al., 2014 Canada [65]	Spleen Lymph nodes	NOD	CD4^−^ CD8^−^	To define the genetic basis underlying differences in the proportion of DNT cells between diabetes-prone and diabetes-resistant mice	FACS [FACSCalibur (BD Biosciences)]	*Lower DNT cell proportion is associated not only with higher diabetes incidence but also with increased serum IgG autoantibody levels, suggesting DN T cells may influence humoral immune regulation.*	*“In summary, studying the genetic underpinnings of immunoregulatory DN T cells has revealed that, as for susceptibility to autoimmune diabetes, it is a complex trait. Interestingly, at least two Idd susceptibility loci are linked to the proportion of DN T cells, emphasizing their relevance in contributing to autoimmune diabetes resistance”*
Liu et al., 2016, China [63]	Spleen Lymph nodes	NOD	CD4^−^ CD8^−^ αβTCR^+^	To investigate the regulation of different subsets of T cells in vivo and in vitro and assess the potential therapeutic implications	FACS [FACSAria (BD Biosciences)]	--	*“…*ex vivo *CD4+ T cell converted DNT cells leads to a long term reversal of new-onset diabetes in NOD mice. […] Combined ATS and DN T cell treatment resulted in significant reversion of new-onset autoimmune diabetes in NOD mice”*
Collin et al., 2018 Canada [67]	Spleen Lymph nodes [Thymus]	NOD	CD4^−^ CD8^−^	To explore the genetic factors that can influence the proportion of the DNT cells, with specific regard to Idd2 and Idd13 loci	FACS [FACSCanto, LSRII, Fortessa x-20 FACSCalibur (BD Bioscencies)]	--	*“…we find that genetic interactions between Idd2 and Idd13 loci modulate cell cycle progression, which contributes, at least in part, to defining the proportion of DN T cells in secondary lymphoid organs.”*
Collin et al., 2021, Canada [68]	Spleen Lymph nodes	NOD	CD4^−^ CD8^−^	To explore the impact of a chromosome 12 locus in autoimmune diabetes, with specific regard to DN T cells	FACS [n/a]	--	*“…this study identified further complex* *genetic interactions in defining the proportion of DN T cells, along with evidence of genetic epistasis within a locus on chromosome 12 influencing autoimmune susceptibility.”*
Islam et al., 2025, USA [69]	Blood Pancreas (islets)	NOD	CD8^−^ CD4^−^ NK1.1^−^ [αβTCR] [γδTCR]	To identify any T-cell clonal expansion and specific transcriptomic signatures, if any, associated with diabetic progression	FACS [Attune NxT (Invitrogen)] Analysis of single-cell gene expression	--	*“…diabetic mice were found to have shockingly high levels of circulating and invading DN T cells and increased exhaustion of potentially immunosuppressive T cell subsets. DN T cell subsets increased during diabetogenesis suggesting that they proliferate more, die less, or are trafficked out of the pancreas more in concert with islet destruction.”*

Abbreviations: NOD—Non-obese diabetic mouse; DP—diabetes-prone; P14—express transgenic TCR(Vα2) specific for the gp33 peptide; RIP-gp—rat insulin promoter transgenic mouse expressing gp33 on the pancreatic β-islet cells; P14/RIP-gp—double transgenic mouse bred from P14 and RIP-gp mice; NOD-gld/—FasL haploinsufficient NOD mouse; CD—cluster differentiation; TCR—T-cell receptor; FACS—Fluorescence-Activated Cell Sorting; Idd—insulin-dependent diabetes loci; n/a—not available (information).

## Data Availability

Not applicable.
